# Strategies to increase couples HIV testing and counselling in sub‐Saharan Africa: a systematic review

**DOI:** 10.1002/jia2.26075

**Published:** 2023-03-16

**Authors:** Karen M. Hampanda, Krysta Pelowich, Kellie Freeborn, Lauren A. Graybill, Wilbroad Mutale, Katelyn R. Jones, Friday Saidi, Andrew Kumwenda, Margaret Kasaro, Nora E. Rosenberg, Benjamin H. Chi

**Affiliations:** ^1^ Department of Obstetrics and Gynecology University of Colorado Anschutz Medical Campus Aurora Colorado USA; ^2^ Center for Global Health University of Colorado Anschutz Medical Campus Aurora Colorado USA; ^3^ Department of Obstetrics and Gynecology School of Medicine University of North Carolina at Chapel Hill Chapel Hill North Carolina USA; ^4^ Department of Epidemiology Gillings School of Global Public Health University of North Carolina at Chapel Hill Chapel Hill North Carolina USA; ^5^ Department of Health Policy School of Public Health University of Zambia Lusaka Zambia; ^6^ Department of Health Behavior Gillings School of Global Public Health University of North Carolina at Chapel Hill Chapel Hill North Carolina USA; ^7^ UNC Project Malawi Lilongwe Malawi; ^8^ Department of Obstetrics and Gynecology School of Medicine University of Zambia Lusaka Zambia; ^9^ UNC Global Projects Zambia Lusaka Zambia

**Keywords:** CHTC, dyads, heterosexual couples, HIV testing, interventions, partners

## Abstract

**Introduction:**

Couple HIV testing and counselling (CHTC) is associated with measurable benefits for HIV prevention and treatment. However, the uptake remains limited in much of sub‐Saharan Africa, despite an expanded range of strategies designed to promote access.

**Methods:**

Following PRIMSA guidelines, we conducted a systematic review to characterize CHTC uptake strategies. Five databases were searched. Full‐text articles were included if they were: conducted in sub‐Saharan Africa during the study period (1980–2019), targeted heterosexual couples, reported at least one strategy to promote CHTC and provided a quantifiable measure of CHTC uptake. After the initial and full‐text screening, key features of the studies were abstracted and synthesized.

**Results:**

Of the 6188 unique records found in our search, 365 underwent full‐text review with 29 distinct studies included and synthesized. Most studies recruited couples through antenatal care (*n* = 11) or community venues (*n* = 8) and used provider‐based HIV testing (*n* = 25). The primary demand creation strategies included home‐based CHTC (*n* = 7); integration of CHTC into clinical settings (*n* = 4); distribution of HIV self‐testing kits (*n* = 4); verbal or written invitations (*n* = 4); community recruiters (*n* = 3); partner tracing (*n* = 2); relationship counselling (*n* = 2); financial incentives (*n* = 1); group education with CHTC coupons (*n* = 1); and HIV testing at other community venues (*n* = 1). CHTC uptake ranged from negligible to nearly universal.

**Discussion:**

We thematically categorized a diverse range of strategies with varying levels of intensity and resources used across sub‐Saharan Africa to promote CHTC. Offering CHTC within couples’ homes was the most common approach, followed by the integration of CHTC into clinical settings. Due to heterogeneity in study characteristics, we were unable to compare the effectiveness across studies, but several trends were observed, including the high prevalence of CHTC promotion strategies in antenatal settings and the promising effects of home‐based CHTC, distribution of HIV self‐tests and integration of CHTC into routine health services. Since 2019, an updated literature search found that combining partner notification and secondary distribution of HIV self‐test kits may be an additionally effective CHTC strategy.

**Conclusions:**

There are many effective, feasible and scalable approaches to promote CHTC that should be considered by national programmes according to local needs, cultural context and available resources.

## 1 INTRODUCTION

In sub‐Saharan Africa—the epicentre of the HIV pandemic—two‐thirds of incident HIV infections occur among stable couples [1]. Couples HIV testing and counseling (CHTC) is an important gateway to mutual status disclosure within a couple, identification of sero‐different partners, HIV risk management, and linkage to HIV care and treatment for those living with HIV. CHTC offers numerous public health benefits [2]; interventions targeting the couple as a unit can increase social support, healthy relationship skills and relationship satisfaction, reduce conflict and stigma, and promote shared decision‐making regarding sexual and reproductive health [3, 4, 5]. In fact, couples‐based interventions may be more effective in promoting HIV protective behaviours than interventions delivered to individuals [6, 7]. CHTC is considered a cost‐effective intervention recommended by the World Health Organization with the potential to avert more than 50% of new HIV infections in sub‐Saharan Africa [8, 9].

Over the past several decades, the number of approaches to promote CHTC has rapidly expanded in sub‐Saharan Africa. CHTC is now available in most clinical settings, as well as community or home‐based settings [10, 11]. Couple members may present together at a clinic appointment for simultaneous HIV testing, be tested separately but brought together for post‐test counselling, or given HIV self‐test kits for use as a couple at home [12]. There is a large range of individual and community‐wide promotion strategies, such as partner invitations, partner tracing, community mobilization, incentives and various other behavioural interventions [13—16].

Despite its clear benefits and various access points, CHTC remains under‐utilized by couples in sub‐Saharan Africa [17]. A recent meta‐analysis found that fewer than one‐third of participants across 14 studies in the region underwent CHTC when offered [18]. To date, there has not been a systematic examination or classification of the wide range of strategies used at the individual or community level—including both randomized trials and larger‐scale programme evaluations—to promote CHTC in sub‐Saharan Africa, and the extent to which these approaches effectively increase CHTC uptake. In this systematic review, we seek to address this gap.

## 2 METHODS

### 2.1 Search strategy and eligibility criteria

This systematic review is registered with PROSPERO (CRD42018086844). Using a comprehensive search strategy developed in collaboration with a reference librarian (Supplementary File), we searched Cochrane Central Register of Controlled Trials (CENTRAL), CINAHL, EMBASE, PsycInfo and PubMed for studies describing strategies to promote the uptake of CHTC in sub‐Saharan Africa. Articles were included if they met the following criteria: (1) described a strategy for increasing the uptake of couple HIV testing or post‐test couple counselling conducted in sub‐Saharan Africa; (2) based on primary data collection; (3) unique full‐text article in a peer‐reviewed journal between 1 January 1980 and 30 September 2019; (4) reported at least one quantifiable measure of couple or partner HIV counselling or testing uptake; (5) temporality between the strategy and uptake of couple testing was reported or could be inferred; (6) focused on heterosexual couples in a past or current sexual relationship; and (7) published in English.

We characterized CHTC broadly. Most articles described CHTC as simultaneous testing and counselling of both partners in a couple conducted by a trained healthcare worker. However, we defined CHTC to include post‐test counselling or disclosure support following both couple members individually testing, or as client report of self‐testing with their partner. We recognize that this expands traditional definitions of CHTC, but allowed for us to include important innovations, such as HIV self‐testing and index tracing approaches, which are prominent in recent policy discussions.

### 2.2 Study screening and selection

Search results were downloaded to EndNote (Clarivate, Philadelphia, PA, USA) and duplicates were removed. The EndNote library was then uploaded to Covidence (Veritas Health Innovation, Melbourne, Australia), an internet‐based software program used for study screening in systematic reviews. All titles and abstracts were double‐screened to identify a pool of potential articles for full‐text review. A calibration exercise was conducted prior to screening to ensure inter‐coder reliability. At least three team members met to discuss discrepant responses until a consensus was reached. The selection phase involved the full‐text review of the articles identified in the screening phase. In the selection phase, the processes of calibration, double review and team reconciliation were repeated to ensure all eligibility criteria were met. Through this staged approach, a final set of articles was identified for data extraction.

### 2.3 Data extraction

Two reviewers independently extracted key features of each study into standardized electronic data collection forms. Characteristics of interest included: the country and years of implementation, study design, study objectives, recruitment site, age of participants (i.e. adults, adolescents or both), a description of how the study defined CHTC (including venue and provider), a description of the strategies implemented to promote CHTC, CHTC uptake measures and any downstream outcomes. Discrepancies were resolved through team discussion until a consensus was reached. If two or more publications reported the same source data, information from both were included in a single record. If two or more publications reported on unique data from the same study, two records were used.

### 2.4 Methodological approach

Given considerable variability in the types of strategies used to promote CHTC uptake, and the diversity of definitions of CHTC used in the literature, meta‐analysis was not feasible [19]. Instead, we conducted a narrative synthesis to describe different strategies used to promote CHTC and their subsequent effect on the uptake of CHTC. As studies often used concurrent strategies to promote CHTC uptake, we categorized studies according to the primary strategy under evaluation reported in the primary source (i.e. described as “the intervention” or the approach emphasized in the article text), and summarized CHTC uptake within these broad groupings. In the results section, we describe whether the study included additional components/approaches and compare this to other studies in the same category. Where possible, we report estimates of effectiveness and provide details on the index and referent groups used in each study's analysis.

### 2.5 Appraisal of study quality

For studies that targeted CHTC as a primary outcome, we appraised study quality using the Mixed Methods Appraisal Tool (MMAT). The MMAT is designed to assess both randomized and non‐randomized quantitative studies [20]. Two reviewers independently assessed quality indicators, including whether randomization was appropriate; study groups were comparable; the sample was representative; measures were appropriate; complete outcome data were included; adherence to assigned interventions was adequate; and confounders were accounted for in the analysis. The MMAT provides a detailed presentation of the ratings of each criterion to inform study quality, rather than an overall score. Studies in this review that reported CHTC as a secondary or exploratory outcome were not included in the quality appraisal given the study design was aimed at testing the effect of an intervention on some other primary outcome.

## 3 RESULTS

### 3.1 Search results

**Figure 1:**
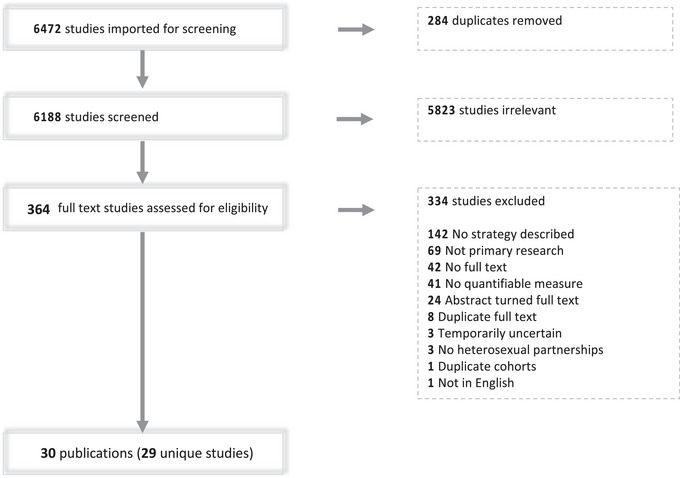
The Preferred Reporting Items for Systematic reviews and Meta‐Analyses (PRISMA) four‐phase flow diagram (records identified, records screened, full texts assessed for eligibility and studies included in review).

Our search identified 6188 unique records. Of these, 5823 were excluded during the title and abstract screening, and 365 full texts were examined for eligibility. After full‐text review, 334 references were ineligible for the following reasons: duplicate article, cohort or analysis (*n* = 33); not English language (*n* = 1); not primary research (*n* = 69); no full text (*n* = 42); no heterosexual partnerships (*n* = 3); no CHTC strategy described (*n* = 142); no quantifiable measure of CHTC uptake (*n* = 41); and uncertain temporality between the implementation of a strategy to promote CHTC uptake and measures of CHTC uptake (*n* = 3). A total of 30 eligible articles reflecting 29 unique studies were included in data abstraction (Figure 1).

### 3.2 Study characteristics

The majority of studies were implemented between 2001 and 2010 (*n* = 10) or after 2010 (*n* = 17). Studies were conducted in the Democratic Republic of the Congo (formerly Zaire; *n* = 2); Kenya (*n* = 9); Malawi (*n* = 2); South Africa (*n* = 3); Tanzania (*n* = 5); Uganda (*n* = 4); Zambia (*n* = 1); and Zimbabwe (*n* = 1). In addition, two studies reported results from two countries: Rwanda and Zambia [21, 22, 23]. Seventeen studies reported CHTC uptake as a primary outcome; the remaining 12 measured CHTC uptake as a secondary or exploratory outcome (Table 1).

**Table 1:** Characteristics of 30 published articles from 29 unique studies included in the systematic review for couples HIV counselling and testing, 1993–2019

Citation	Country	Data collection years	Study design	Study population age	Recruitment site	CHTC outcome: primary or secondary	CHTC definition from primary source	Location of CHTC	HIV counselling and/or testing provider
Allen et al. (2007)	Rwanda, Zambia	2003	Cohort	Adults	Other clinic	Primary	Couple counselled and tested together	Antenatal clinic	Study team
Becker et al. (2010)	Tanzania	2003–2004	Randomized controlled trial	Adolescents + adults	Antenatal clinic	Secondary	Couple counselled and tested together, or couple counselled together after individual testing	Antenatal clinic	Study team
Becker et al. (2014)	Malawi	2003	Cohort	Adolescents + adults	Home	Primary	Couple counselled and tested together	Home	Study team
Courtenay‐Quirk et al. (2018)	Tanzania	2014–2015	Cohort	Adolescents + adults	Other clinic	Primary	Couple counselled together after individual testing	Clinic	Clinic staff
Dalal et al. (2013)	Kenya	2008	Cohort	Adolescents + adults	Home	Primary	Couple counselled and tested together	Home	Study team
Darbes et al. (2019)	South Africa	2012–2014	Randomized controlled trial	Adults >18 years only	Mobile testing centre	Primary	Couple counselled and tested together	Community	Study team
Ditekemena et al. (2011)	DRC	2006–2007	Randomized controlled trial	Adults >18 years only	Other clinic, community venue	Secondary	Couple counselled and tested together	Community	Study team
Doherty et al. (2013)	South Africa	2009–2010	Cluster randomized controlled trial	Adolescents + adults	Home	Secondary	Couple counselled and tested together	Home	Study team
Gichangi et al. (2018)	Kenya	2015–2016	Randomized controlled trial	Adults >18 years only	Antenatal clinic, home	Primary	Couple tested together	Clinic or home	Self or clinic staff
Heyward et al. (1993)	Zaire (now, DRC)	1989–1991	Cohort	Adolescents + adults	Antenatal clinic, home	Secondary	Couple counselled together after individual testing	Clinic	Clinic staff
Homsy et al. (2006)	Uganda	2004–2005	Cohort	Adolescents + adults	Antenatal clinic, other clinic	Secondary	Couples counselling and testing together or couples counselling together after individual testing	Antenatal clinic	Clinic staff
Jefferys et al. (2015)	Tanzania	2013	Cohort	Adolescents + adults	Antenatal clinic	Secondary	Couple counselled and tested together	Antenatal clinic	Clinic staff
Joseph Davey et al. (2019)	South Africa	2017–2018	Cohort	Adolescents + adults	Antenatal clinic, other clinic	Secondary	Couple counselled and tested together or couple counselled together after individual testing	Clinic	Clinic staff
Kababu et al. (2018)	Kenya	2014	Quasi experimental (pre‐/post‐implementation)	Adolescents + adults	Other clinic	Primary	Couple counselled and tested together	Clinic	Clinic staff
Krakowiak et al. (2016)	Kenya	2013–2014	Pragmatic trial	Adolescents + adults	Antenatal clinic, home	Primary	Couple counselled and tested together or couple counselled together after individual testing	Antenatal clinic	Clinic staff
Lambdin et al. (2011)	Zambia	2004–2006	Cohort	Adolescents + adults	Other clinic	Primary	Couple counselled and tested together	Community	Study team
Lyatuu et al. (2018)	Tanzania	2015–2016	Non‐randomized implementation evaluation study	Adolescents + adults	Antenatal clinic	Primary	Couple counselled and tested together	Antenatal clinic	Clinic staff
Masters et al. (2016)	Kenya	2015–2016	Randomized controlled trial	Adults >18 years only	Antenatal clinic, home	Secondary	Couple tested together	Home	Self
Matovu et al. (2002)	Uganda	1999–2000	Cohort	Adolescents + adults	Other clinic, home	Secondary	Couple counselled and tested together	Home	Study team
Matovu et al. (2016)	Uganda	2014	Cluster randomized controlled trial	Adolescents + adults	Other clinic, home	Primary	Couple counselled and tested together	Community‐based location or clinic	Study team or clinic staff
Osoti et al. (2014)	Kenya	2012–2013	Randomized controlled trial	Adults >18 years only	Antenatal clinic, home	Primary	Couple counselled and tested together	Home or clinic	Study team or clinic staff
Pintye et al. (2019)	Kenya	2017–2018	Cohort	Adolescents + adults	Other clinic, home	Secondary	Couple tested together	Home	Self
Rosenberg et al. (2015)	Malawi	2014	Randomized controlled trial	Adolescents + adults	Antenatal clinic	Primary	Couple counselled and tested together	Antenatal clinic	Clinic staff
Sibanda et al. (2017)	Zimbabwe	2015–2016	Cluster randomized controlled trial	Adolescents + adults	Mobile testing centre	Primary	Couple counselled and tested together	Community location/mobile location	Study team
Theuring et al. (2016)	Tanzania	2013	Controlled intervention trial	Adults >18 years only	Antenatal clinic	Primary	Couple counselled and tested together	Antenatal clinic	Clinic staff
Thirumurthy et al. (2016)	Kenya	2015	Cohort	Adults > 18 years only	Antenatal clinic, other clinic, home	Secondary	Couple tested together	Antenatal clinic or other clinic	Self or clinic staff
Tumwebaze et al. (2012)	Uganda	2010–2011	Cohort	Adults > 18 years only	Home	Secondary	Couple counselled together after individual testing	Home	Study team
Turan et al. (2018)	Kenya	2014–2017	Randomized controlled trial	Adults > 18 years only	Antenatal clinic, home	Primary	Couple counselled and tested together	Antenatal clinic	Clinic staff
Wall et al. (2012a), Wall et al. (2012b)	Zambia Rwanda	2004–2005	Cohort	Adolescents + adults	Other clinic, mobile testing centre	Primary	Couple counselled and tested together	Community location/mobile location	Study team

Abbreviations: CHTC, couples HIV testing and/or counselling; DRC, Democratic Republic of the Congo.

There was considerable variability with respect to study design, study population and recruitment site. Most studies (*n* = 14) were observational cohort studies. Eight studies were individual randomized controlled trials and three were cluster‐randomized controlled trials. Four used other designs. Nineteen studies enrolled both adults and adolescents, while 10 enrolled adults only. Studies often used multiple recruitment sites, including antenatal clinics (*n* = 17); clinics outside of antenatal care (*n* = 8); homes (*n* = 14); and other community venues (*n* = 3).

The definition of CHTC and the location of CHTC varied across studies. In most studies (*n* = 18), CHTC was defined as couples counselled and tested for HIV together, simultaneously. Other studies defined CHTC as couples counselled together after individual testing (*n* = 3); either counselling and testing together or counselling together after individual testing (*n* = 4); or couples tested together (either by a provider or with self‐testing) with no mention of counselling (*n* = 4). CHTC occurred at antenatal clinics (*n* = 20), homes (*n* = 18), community‐based or mobile locations (*n* = 12) or other clinic types (*n* = 14). CHTC was most frequently conducted by a provider (*n* = 25), though some studies used self‐testing (*n* = 2) or a choice of self or provider testing (*n* = 2).

Studies employed numerous strategies to promote CHTC at the individual, health facility and community levels, including combinations of approaches. We grouped these into 10 categories based on the main CHTC approach: home‐based CHTC (*n* = 7); enhancing the clinic environment with integrated CHTC (*n* = 4); verbal or written invitation (*n* = 4); distribution of HIV self‐testing kits (*n* = 3); community agents/recruiters (*n* = 3); index partner tracing (*n* = 2); intensive couple counselling to strengthen relationships (*n* = 2); financial incentives (*n* = 1); group education with CHTC invitation coupons (*n* = 1); and offering HIV testing at other community venues (*n* = 1). Each category is described in detail below, alongside observed trends in CHTC uptake. Full details are provided in Table S1.

### 3.3 Home‐based couples HIV counselling and testing

The most common approach, home‐based testing, resulted in CHTC uptake ranging from 17% to 87%. Four studies using home‐based CHTC (along with other interventions) did not have a comparison arm. In Tanzania, Becker et al. found that joint home‐based CHTC along with family planning services resulted in the highest reported uptake of CHTC (87%) in this category [24]. Studies of door‐to‐door outreach for CHTC (21–52%) [11, 25, 26] or as part of epidemiological survey research (17%) [27] were also reported, with lower levels of success. Three studies offering home‐based CHTC had a comparison arm of either the standard of care (HIV counselling and testing services at local clinics) [26] or written invitations given in antenatal clinics for male partners to attend the clinic [28, 29]. In all three studies, couples in the home‐based groups had considerably higher uptake of CHTC compared to the control group (21% vs. 10%; 77% vs. 24%; and 85% vs. 36%) [26, 28, 29].

### 3.4 Enhanced clinic environment

Four observational cohort studies used some form of clinic enhancement to promote CHTC. Two of the studies integrated CHTC services into antenatal clinics [30, 31]. After integrating opt‐out HIV counselling and testing services into maternal–child health services in Uganda, Homsy et al. found that couples made up only 3% of those testing for HIV in antenatal clinics and 37% of those testing in the maternity ward (postpartum care); however, CHTC was not the primary outcome of the study. Conversely, in a larger study of over 7000 participants in Tanzania, Lyatuu et al. sought to increase male partner participation by offering couple‐friendly services at antenatal clinics and promoting male partner participation via community‐led activities. After 1 year, couple HIV testing in the intervention sites had tripled from 12% at baseline to 36%, with very little change in the control sites (approximately 18% both pre‐ and post‐implementation) [30]. Outside of antenatal clinics, Kababu et al. integrated a counsellor‐supported disclosure model to empower the index HIV testing client to invite their sexual partner for CHTC at the clinic and link them to post‐HIV test interventions in Kenya. Participants who received the counsellor‐supported disclosure model had a significantly higher uptake of CHTC (28%) compared to the standard of care control group (7%) [12]. Lastly, Courtenay‐Quirk et al. found that enhancing record‐keeping of HIV service delivery and training clinic staff and peer educators on CHTC improved the uptake of CHTC among newly diagnosed tuberculosis patients in Tanzania. When implemented in two clusters (i.e. immediate vs. delayed implementation), CHTC rates increased from 2% at baseline to over 35% following implementation [10].

### 3.5 Distribution of HIV self‐test kits

Four studies—all conducted in Kenya—examined whether the distribution of HIV self‐test kits to women at different health facility venues promoted the uptake of CHTC. Thirumurthy et al. provided multiple self‐tests to women at antenatal clinics, postpartum clinics and at a drop‐in centre for female sex workers. The uptake of CHTC was 51%, 68% and 83%, respectively, at these venues [32]. Masters et al. similarly provided two HIV self‐testing kits to women attending antenatal or postpartum care and encouraged them to distribute a test kit to their male partner or use both kits for testing as a couple. Compared to women who received an invitation card for male partner HIV testing, women who received HIV self‐test kits were more likely to report the uptake of CHTC within 3 months (75.4% vs. 33.2%, *p*<0.001) [33]. Pintye et al. also offered HIV self‐tests for at‐home couple or partner HIV testing to women without HIV seeking routine maternal and child health and family planning services at health facilities. In this study, 24% of women accepted self‐test kits. Among those retained in care (44%), most (87%) reported testing together with their partner [34]. Gichangi et al. conducted a three‐arm randomized trial within antenatal settings. They found that CHTC uptake was significantly higher in the HIV self‐test arm (79%), compared to the standard‐of‐care information card arm (27%) and the enhanced information card arm (35%) [35].

### 3.6 Verbal or written invitation letters to male partners

Four studies tested verbal or written invitations to promote CHTC. One study described low rates of CHTC uptake (2%) when women were verbally encouraged to bring in their male partners during their HIV post‐test counselling in Zaire, now the Democratic Republic of the Congo [36]. Three studies—all within the context of antenatal care—used written invitation letters to the male partners of pregnant women to promote CHTC. In two studies from Tanzania, pregnant women brought an invitation letter home, requesting the male partner's presence at the next routine antenatal care visit, without specific mention of HIV testing or CHTC. The proportions of couples tested varied widely from 16% reported by Becker et al. to 81% reported by Jefferys et al. [37, 38]. Notably, comparison groups were not included in either study. In contrast, Theuring et al. compared the uptake of CHTC in Tanzania between women given written invitation letters for their male partners to women who were instructed to verbally invite their partners. The uptake of CHTC was comparable between the two groups (31% vs. 28%, *p* = 0.59) [39]. Five studies from Kenya and one study from Malawi used written invitation letters as the comparison/control arm. In the Kenya studies, the uptake of CHTC among those offered a letter ranged from 24% to 36% [28, 40, 41]. In contrast, Rosenberg et al. reported 52% CHTC uptake in their control arm using invitation letters in Malawi [15].

### 3.7 Community agents and recruiters

Three large observational cohort studies reported on the use of community agents or recruiters to promote CHTC. In each, influential network leaders/agents advertised CHTC throughout various community venues, including in neighbourhoods, churches, parent–teacher associations and places of employment. In two cities in Zambia, Lambdin et al. tested the use of community agents to contact close interpersonal connections, such as friends and neighbours, to encourage CHTC. The intervention also included various forms of media advertisements for CHTC to increase demand. This approach resulted in high CHTC uptake, measured at 71% and 75% in the two sites [42]. These figures are much higher than a different programme in Zambia (6%) or Rwanda (18%) that used community outreach in churches, employment and health clinics [21, 22].

### 3.8 Assisted contact tracing

Two studies reported CHTC uptake following assisted contact tracing. In South Africa, Joseph Davey et al. provided an index testing strategy, whereby clinic staff traced partners and children of index patients who were recently diagnosed with HIV or on HIV treatment. The index patient was offered three options to encourage HIV testing among family members: a written invitation letter, a phone call from the clinician or a return clinic visit with the index patient and family member(s). This patient choice strategy, which was implemented in antenatal and tuberculosis clinics, led to a 40% uptake of CHTC [43]. In a randomized controlled trial, Rosenberg et al. compared two strategies to promote CHTC among male partners of pregnant women recently diagnosed with HIV in Malawi: a written letter inviting male partners to attend the antenatal clinic (control) versus an invitation plus enhanced partner tracing (intervention). After 1 month, more couples who received an invitation plus enhanced partner tracing presented to the clinic for CHTC compared to written invitation only (75% vs. 52%, *p* = 0.001) [44].

### 3.9 Couple counselling to strengthen relationships

Two studies evaluated intensive multi‐session couples counselling interventions designed to strengthen relationships, with the goal of increasing uptake of CHTC. In Kenya, Turan et al. reported that couples who received three counselling sessions and were offered home‐based HIV testing were nearly three times more likely to complete CHTC than male partners who received written invitation letters to attend an antenatal clinic (64% vs. 23%) [41]. In South Africa, Darbes et al. tested an intervention that included one couples‐based group session followed by four individual couple counselling sessions. This approach resulted in higher CHTC uptake compared to written invitations for male partners (42% vs. 12%, *p*<0.001) [45].

### 3.10 Other strategies

Three studies described strategies that did not fall within the above categories. In Zimbabwe, Sibanda et al. found that providing a financial incentive for groceries (equivalent to US$1.50) resulted in greater uptake of CHTC, compared to standard of care (HIV testing via mobile units; 56% vs. 10%) [46]. In Uganda, Matovu et al. reported higher uptake of CHTC among couples who received small group couple and male‐focused interactive sessions and invitation coupons for CHTC compared to couples who received general adult health education sessions (20% vs. 14%; *p* = 0.04) [47]. Finally, in the Democratic Republic of the Congo, Ditekemena et al. conducted a trial where the male partners of pregnant women were randomized to be offered HIV testing at one of three venues: health facility, bar or church. Men who were tested at one of the venues were then invited for couple counselling with their female partners. CHTC attendance from men who were tested at the church venue (46%) and bar venue (38%) was significantly higher compared to the health centre venue (20%; *p*<0.001) [48].

### 3.11 Downstream outcomes

Downstream outcomes were reported by some studies in this review. Several studies found that CHTC promotes linkages to HIV care and treatment among newly diagnosed people with HIV [11, 15, 25, 29, 32, 37, 43]. There was also evidence that CHTC reduces sexual risk behaviours by increasing condom use and reducing outside partners [15, 32, 36, 37, 45]. Other studies found that CHTC increased voluntary medical male circumcision [25] and contraceptive use [36]. Improvements in couple communication [37, 41, 45], couples’ sexual and reproductive health decision‐making [45] and social support were also reported after CHTC [29, 41]. Additionally, studies reported that CHTC was associated with maternal postpartum checkups and exclusive breastfeeding [41], nevirapine use [37] and prevention of mother‐to‐child transmission (PMTCT) care [15]. No negative consequences, such as intimate partner violence, were attributable to CHTC [33, 37, 41, 46] and participants generally reported satisfaction with CHTC and the promotion strategies [41, 45]. One study found that a minority of women with HIV experienced “problems” during mutual disclosure [45].

### 3.12 Quality appraisal

Quality appraisals were performed for 18 studies that measured CHTC as the primary outcome (Table 2). Eleven studies were excluded from the quality appraisal because they reported on CHTC as a secondary or exploratory outcome. All 18 studies included in the quality appraisal posed clear research questions. All but one study collected data that allowed them to address the research questions. Among the eight randomized controlled trials, all performed appropriate randomization, had comparable baseline data and reported complete outcome data (almost all the participants contributed to almost all measures). In contrast, only two reported that assessors were blinded to the intervention provided and only three reported adherence to the assigned intervention. Among the 10 studies with alternate designs, eight had appropriate outcome measures; 10 had complete outcome data; six accounted for confounders in the design and analysis; and seven administered the intervention as intended during the study period.

**Table 2:** Quality appraisal of included studies with couples HIV counselling and testing as primary outcome

Citation	All studies		Randomized controlled trials only					Other study designs only				
	Are there clear research questions?	Do the collected data allow to address the research questions?	Is randomization appropriately performed?	Are the groups comparable at baseline?	Are there complete outcome data?	Are outcome assessors blinded to the intervention provided?	Did the participants adhere to the assigned intervention?	Are the participants representative of the target population?	Are measurements appropriate regarding both the outcome and exposure/intervention?	Are there complete outcome data?	Are the confounders accounted for in the design and analysis?	During the study period, is the intervention/ exposure administered as intended?
Darbes et al. (2019)	Yes	Yes	Yes	Yes	Yes	Unknown	Yes	—	—	—	—	—
Gichangi et al. (2018)	Yes	Yes	Yes	Yes	Yes	Yes	Unknown	—	—	—	—	—
Matovu et al. (2016)	Yes	Yes	Yes	Yes	Yes	No	Unknown	—	—	—	—	—
Osoti et al. (2014)	Yes	Yes	Yes	Yes	Yes	Yes	Unknown	—	—	—	—	—
Rosenberg et al. (2015)	Yes	Yes	Yes	Yes	Yes	No	Unknown	—	—	—	—	—
Sibanda et al. (2017)	Yes	Yes	Yes	Yes	Yes	No	Unknown	—	—	—	—	—
Theuring et al. (2016)	Yes	Yes	Yes	Yes	Yes	No	Yes	—	—	—	—	—
Turan et al. (2018)	Yes	Yes	Yes	Yes	Yes	Unknown	Yes	—	—	—	—	—
Allen et al. (2007)	Yes	Yes	—	—	—	—	—	Yes	Yes	Yes	Unknown	Yes
Becker et al. (2014)	Yes	Yes	—	—	—	—	—	Unknown	Yes	Yes	Yes	Unknown
Courtenay‐Quirk et al. (2018)	Yes	Yes	—	—	—	—	—	Yes	Yes	Yes	No	Yes
Dalal et al. (2013)	Yes	Yes	—	—	—	—	—	Yes	Yes	Yes	Yes	Yes
Kababu et al. (2018)	Yes	Yes	—	—	—	—	—	Unknown	Yes	Yes	Yes	Yes
Krakowiak et al. (2016)	Yes	Yes	—	—	—	—	—	Yes	Yes	Yes	Unknown	Unknown
Lambdin et al. (2011)	Yes	Yes	—	—	—	—	—	Yes	Yes	Yes	No	Unknown
Lyatuu et al. (2018)	Yes	Unknown	—	—	—	—	—	Yes	Yes	Yes	Yes	Yes
Wall et al. (2012a)	Yes	Yes	—	—	—	—	—	Yes	Yes	Yes	Yes	Yes
Wall et al. (2012b)	Yes	Yes	—	—	—	—	—	Yes	Yes	Yes	Yes	Yes
Total	18/18 (100%)	17/18 (94%)	8/8 (100%)	8/8 (100%)	8/8 (100%)	2/8 (25%)	3/8 (37.5%)	8/10 (80%)	10/10 (100%)	10/10 (100%)	6/10 (60%)	7/10 (70%)

## 4 DISCUSSION

In this systematic review, we identified 29 unique studies across nine African countries that described one or more approaches to promote CHTC uptake among heterosexual couples. A diverse range of strategies were evaluated and a wide range of CHTC uptake was observed. We described the various interventions that have been used to promote CHTC in sub‐Saharan Africa and mapped them thematically according to approach. Our work provides an organizing framework, derived from empiric data, to guide future policy and programmatic discussions of CHTC promotion. As national HIV programmes move towards ambitious global targets to end the HIV epidemic [50], understanding different CHTC approaches—and their supporting evidence base—is essential.

Although other systematic reviews have examined individual HIV testing, male partner engagement and couples‐based interventions for HIV [51, 52, 53, 54, 55], the scope of our current analysis is a distinct and valuable complement. We were deliberately inclusive with our eligibility criteria, to ensure that we captured the full scope of CHTC approaches published in the literature. This stands in contrast to Hailemariam and colleagues, who estimated the uptake of CHTC across 14 studies in sub‐Saharan Africa. In that systematic review and meta‐analysis, studies were included only if they provided an estimate of the proportion or number of couples in a heterosexual relationship who undertook CHTC in sub‐Saharan Africa between 2000 and 2017 [18]. Their meta‐analysis provided a pooled estimate for CHTC across the region (31.5%, 95% CI: 23.6–40.0%) but with fewer source studies compared to the present review (12 vs. 29) due to narrower inclusion criteria [18]. Moreover, these authors examined the uptake of CHTC by several study characteristics, but did not describe or compare the specific approaches used to promote CHTC.

Among our included studies, we observed heterogeneity in outcome definitions. While most studies defined CHTC as both members of the couple testing and counselled *together*, this was not universal. Some studies considered only one component (i.e. either testing or counselling), while others focused on proxies, such as mutual HIV status disclosure. When measuring the relative success of the strategy, differences in the analytic approach—more specifically, the denominator of the predefined outcome—also made comparisons between studies challenging. Unsurprisingly, the use of different denominators in studies testing similar CHTC strategies (e.g. the number of women who were *given* a written invitation for their male partners vs. the number of pregnant women who *returned* with their male partners to undergo CHTC) resulted in highly varied rates of CHTC uptake [37, 38].

Nearly, half of the included studies focused on pregnant populations in antenatal settings, which is not surprising given the emphasis on male partner involvement in antenatal care and PMTCT throughout sub‐Saharan Africa [56]. Similarly, in a prior review, 23% of included studies were among pregnant couples who had higher rates of CHTC uptake, compared to unspecified couples (38% vs. 27%) [18]. Targeting CHTC during and after pregnancy can leverage the social and cultural expectations about male partner involvement during pregnancy, as well as improved health behaviours observed during this period [57, 58, 59]. The potential benefits for CHTC during pregnancy—and downstream HIV prevention and treatment—should be framed at antenatal care within a family context [60], one that extends beyond the individual.

The evidence on strategies to promote CHTC among adolescents and non‐traditional sexual partnerships from studies in this review was lacking. Few studies in this review included adolescents and none focused exclusively on adolescents. This represents an important gap given the undue burden of HIV in female adolescents and their poor HIV prevention and treatment outcomes [17, 61]. Future research is needed for this group, who experience different challenges, obstacles and social influences than adults. Additionally, most studies required participants to be committed primary partners, married or in a cohabitating relationship. A challenge to home‐based CHTC approaches identified by studies in this review is that a large proportion of individuals have sexual partners who do not live in the same household [25]. Only one study explicitly focused on non‐traditional couples who have high rates of non‐disclosure and low rates of CHTC [45].

Multiple concurrent strategies were often used within one study and even within one study arm. For instance, community recruiters along with improving the clinic environment through couple‐friendly services at antenatal clinics increased couple HIV testing in one study; but it is unclear whether the community recruiters or the improved clinic atmosphere was responsible for the increase [30]. Similarly, home‐based CHTC was paired with intensive couple counselling in another study, which led to increased CHTC uptake; but there is no ability to determine whether the counselling or offering home‐based testing is responsible for the increased uptake of CHTC [41]. In future CHTC research, we recommend using factorial designs, sequential multiple assignment randomized trials (SMART) or other adaptive designs to better understand what approaches are the key ingredients for promoting CHTC uptake among particular groups. Based on the abundance of different strategies proven to effectively promote CHTC in this review, we recommend less focus on developing new CHTC strategies, and rather, focus on expanding evidence‐based approaches.

This review included a mix of larger evaluation studies and effectiveness trials with varying levels of external validity. Studies that evaluated the integration of CHTC into routine healthcare, such as tuberculosis clinics or antenatal/postpartum/maternal and child health care, report this is an effective and low‐burden approach to increase CHTC in various contexts [10, 12, 62]. Similarly, the influence network agent model—tested in several large implementation evaluation studies—was found to be effective, feasible and likely applicable across various other settings to educate and encourage couples to attend CHTC [21, 22, 42]. Some of the intervention trials, including home‐based CHTC and couples counselling strategies, attempted to increase external validity by using, for example, lay counsellors paid typical salaries or were conducted within conditions reflective of local resources [26, 41, 45].

Overall, there remains an ongoing need to better evaluate the generalizability of specific CHTC strategies within and across sub‐Saharan African countries. For instance, while nine African countries were represented in this review, none of the studies took place in West Africa, which lags behind Southern and Eastern Africa in terms of meeting global HIV targets [17]. Certain CHTC strategies, such as HIV self‐testing kits, also only have evidence on effectiveness from one country setting. Some approaches, such as small monetary incentives and group education with HIV testing coupons, came only from rural settings and may have little applicability to urban contexts [46, 47, 49].

We note several limitations to our current study, including that several years have elapsed since our search was conducted and that the literature around CHTC continues to grow. However, among the articles we identified since September 2019, very few would meet our inclusion criteria for various reasons: study protocols [62, 63], secondary data analyses of studies already included [64, 65] and modelling cost‐effectiveness studies [66, 67]. One study, however, adds new evidence that partner notification plus secondary distribution of HIV self‐test kits to pregnant women resulted in greater male partner HIV testing of any sort but reduced male partner facility‐based HIV testing, compared to partner notification services only [68]. The authors conclude that there is a need for stronger support services to link male partners to facilities after self‐testing [68]. While outside the inclusion criteria of our review, recent evidence also indicates that integration of CHTC into ART clinics, distribution of HIV self‐test kits and index partner tracing are cost‐effective approaches to promote CHTC [66, 67, 69]. There is a gap in the literature, however, testing the cost‐effectiveness of other specific CHTC strategies found in this review, which is an important consideration for national HIV programmes and the feasibility of larger implementation.

While critical, HIV testing represents only the first step in the continuum for HIV prevention, care and treatment. A number of studies have shown the attrition that occurs between HIV testing of any sort—including CHTC—and linkages to HIV services, including those for antiretroviral therapy [71]. In this review, we found some evidence that CHTC can promote positive downstream outcomes, including linkages to HIV care and treatment, reduced sexual risk behaviours, male circumcision and contraception uptake, and some maternal and child health outcomes (e.g. PMTCT and breastfeeding). Moving forward, better measurement of these downstream outcomes is critical to understand the full impact and cost‐effectiveness of different CHTC strategies. For example, there is a need to follow newly diagnosed individuals through CHTC beyond a referral to determine whether they sought care and initiated antiretroviral therapy.

## 5 CONCLUSIONS

In summary, this systematic review identified a wide range of approaches used throughout sub‐Saharan Africa over the last several decades to promote CHTC uptake. We were able to characterize the approaches into 10 overarching categories. Due to the heterogeneity in study designs, outcome definitions and source populations, direct comparisons between the different approaches proved difficult. However, several promising approaches and trends were identified, particularly the integration of CHTC into routine health services, home‐based CHTC and HIV self‐testing. Varying source populations among studies highlights opportunities for partner engagement across various groups and health services. As national programmes seek to expand CHTC—a critical component to comprehensive HIV services—selection of specific approaches should consider the evidence supporting the effective promotion of CHTC, along with available resources, existing health infrastructure and cultural context.

## COMPETING INTERESTS

The authors declare no competing interests.

## AUTHORS’ CONTRIBUTIONS

The authors contributed to the following: study idea and conceptual scheme (NER, BHC and LAG), study screening (KMH, KF, LAG, KRJ, NER and BHC), full‐text review (KMH, KF, LAG, NER and BHC), data abstraction (KMH, KF and KP), designing analytic categories (KMH, KP, KF, LAG, NER and BHC), data analysis and interpretation (KMH, LAG, NER and BHC), writing first drafts (NER and KMH) and substantive editing (KMH, NER, BHC, LAG, WM, FS, AK and MK).

## FUNDING

This study was funded by the National Institute of Allergy and Infectious Disease (NIAID) through award R01 AI131060. Additional investigator, trainee and administrative support was provided by NIAID (K24 AI120796, P30 AI50410), National Institute of Mental Health (K99 MH116735, R00 MH104154) and the Fogarty International Center (D43 TW009340, D43 TW010558, D43 TW010060).

## DISCLAIMER

The content is solely the responsibility of the authors and does not necessarily represent the official views of the National Institutes of Health.

## Supporting information

Supplementary information

Supplementary information

## Data Availability

The data extracted for this systematic review are available within the article and the Supplementary Materials.
