# Supplementary material for: Strategies to increase couples HIV testing and counselling in sub‐Saharan Africa: a systematic review
Source: J Int AIDS Soc. 2023 Mar 16;26(3):e26075. doi: 10.1002/jia2.26075 (PMC10020817; doi:10.1002/jia2.26075)
Supplement: Supplementary file 1 — Supplementary information [file JIA2-26-e26075-s001.docx]

**Search Strategy - Strategies to increase couples HIV testing and counseling in sub-Saharan Africa: a systematic review**

Performed 01 Oct 2019

Total records found: 11,154

Records after duplicates removed: 6,188

| **Cochrane Library** – 01 Oct 2019 (3:59pm ET) | | |
| --- | --- | --- |
| **Number** | **Query** | **Results Returned** |
| 1 | MeSH descriptor: [HIV] this term only | 791 |
| 2 | MeSH descriptor: [HIV Infections] this term only | 17,286 |
| 3 | "HIV" or "Human Immunodeficiency Virus" or "Human Immunodeficiency Viruses" or "HIV/AIDS" or "HIV infection" or "HIV infections":ti,ab,kw (Word variations have been searched) | 43893 |
| 4 | #1 or #2 or #3 | 43893 |
| 5 | MeSH descriptor: [Spouses] this term only | 631 |
| 6 | MeSH descriptor: [Marriage] this term only | 537 |
| 7 | "couple" or "couples" or "partner" or "partners" or "partnership" or "partnerships" or "relationship" or "relationships" or "spouse" or "spouses" or "dyad" or "dyads" or "married" or "marital" or "marriage":ti,ab,kw (Word variations have been searched) | 182225 |
| 8 | #5 or #6 or #7 | 182225 |
| 9 | MeSH descriptor: [Counseling] this term only | 7872 |
| 10 | "testing" or "test" or "tests" or "tested" or "screen" or "screening" or "screenings" or "screened" or "counseling" or "counselling" or "counsel":ti,ab,kw (Word variations have been searched) | 670880 |
| 11 | #9 or #10 | 670880 |
| 12 | MeSH descriptor: [Africa South of the Sahara] explode all trees | 5791 |
| 13 | "sub-Saharan Africa" or "Subsaharan Africa" or "sub Saharan Africa" or "sub-Sahara" or "Central Africa" or "Southern Africa" or "East Africa" or "Eastern Africa" or "West Africa" or "Western Africa" or "Angola" or "Benin" or "Botswana" or "Burkina Faso" or "Burundi" or "Cameroon" or "Cape Verde" or "Central African Republic" or "Chad" or "Comoros" or "Congo" or "Cote d Ivoire" or "Ivory Coast" or "Djibouti" or "DRC" or "Eritrea" or "Ethiopia" or "Gabon" or "Gambia" or "Ghana" or "Guinea" or "Kenya" or "Lesotho" or "Liberia" or "Madagascar" or "Malawi" or "Mali" or "Mauritania" or "Mauritius" or "Mayotte" or "Mozambique" or "Mocambique" or "Namibia" or "Niger" or "Nigeria" or "Réunion" or "Rwanda" or "Sahel" or "Sao Tome and Principe" or "Senegal" or "Seychelles" or "Sierra Leone" or "Somalia" or "South Africa" or "RSA" or "Sudan" or "Swaziland" or "Tanzania" or "Togo" or "Uganda" or "Zambia" or "Zimbabwe":ti,ab,kw (Word variations have been searched) | 34558 |
| 14 | #12 or #13 | 34621 |
| 15 | #4 and #8 and #11 and #14 | **989** |

| **CINAHL -** Searched on 01 Oct 2019 (2:25pm) | | |
| --- | --- | --- |
| **Number** | **Query** | **Results Returned** |
| 1 | (MH "Human Immunodeficiency Virus") | 8,389 |
| 2 | (MH "HIV infections") | 114,264 |
| 3 | TI ( "HIV" OR "Human Immunodeficiency Virus" OR "Human Immunodeficiency Viruses" OR "HIV/AIDS" OR "HIV infection" OR "HIV infections" ) OR AB ( "HIV" OR "Human Immunodeficiency Virus" OR "Human Immunodeficiency Viruses" OR "HIV/AIDS" OR "HIV infection" OR "HIV infections" ) | 154,581 |
| 4 | S1 or S2 or S3 | 180,657 |
| 5 | (MH "sexual partners") | 14,704 |
| 6 | (MH "spouses") | 17,572 |
| 7 | (MH "married women") | 1,138 |
| 8 | (MH "married men") | 313 |
| 9 | (MH "marriage") | 14,378 |
| 10 | TI ( "couple" OR "couples" OR "partner" OR "partners" OR "partnership" OR "partnerships" OR "relationship" OR "relationships" OR "spouse" OR "spouses" OR "dyad" OR "dyads" OR "married" OR "marital" OR "marriage" ) OR AB ( "couple" OR "couples" OR "partner" OR "partners" OR "partnership" OR "partnerships" OR "relationship" OR "relationships" OR "spouse" OR "spouses" OR "dyad" OR "dyads" OR "married" OR "marital" OR "marriage" ) | 648,723 |
| 11 | S5 OR S6 OR S7 OR S8 OR S9 OR S10 | 413,571 |
| 12 | (MH "counseling") | 48,726 |
| 13 | TI ( "testing" OR "test" OR "tests" OR "tested" OR "screen" OR "screening" OR "screenings" OR "screened" OR "counseling" OR "counselling" OR "counsel" ) OR AB ( "testing" OR "test" OR "tests" OR "tested" OR "screen" OR "screening" OR "screenings" OR "screened" OR "counseling" OR "counselling" OR "counsel" ) | 1,101,329 |
| 14 | S12 OR S13 | 638,871 |
| 15 | (MH "Africa South of the Sahara+") | 108,713 |
| 16 | TI ( “sub-Saharan Africa” OR “Subsaharan Africa” OR “sub Saharan Africa” OR “sub-Sahara” OR “Central Africa” OR “Southern Africa” OR “East Africa” OR “Eastern Africa” OR “West Africa” OR “Western Africa” OR “Angola” OR “Benin” OR “Botswana” OR “Burkina Faso” OR “Burundi” OR “Cameroon” OR “Cape Verde” OR “Central African Republic” OR “Chad” OR “Comoros” OR “Congo” OR “Cote d Ivoire” OR “Ivory Coast” OR “Djibouti” OR “DRC” OR “Eritrea” OR “Ethiopia” OR “Gabon” OR “Gambia” OR “Ghana” OR “Guinea” OR “Kenya” OR “Lesotho” OR “Liberia” OR “Madagascar” OR “Malawi” OR “Mali” OR “Mauritania” OR “Mauritius” OR “Mayotte” OR “Mozambique” OR “Mocambique” OR “Namibia” OR “Niger” OR “Nigeria” OR “Réunion” OR “Rwanda” OR “Sahel” OR “Sao Tome and Principe” OR “Senegal” OR “Seychelles” OR “Sierra Leone” OR “Somalia” OR “South Africa” OR “RSA” OR “Sudan” OR “Swaziland” OR “Tanzania” OR “Togo” OR “Uganda” OR “Zambia” OR “Zimbabwe” ) OR AB ( “sub-Saharan Africa” OR “Subsaharan Africa” OR “sub Saharan Africa” OR “sub-Sahara” OR “Central Africa” OR “Southern Africa” OR “East Africa” OR “Eastern Africa” OR “West Africa” OR “Western Africa” OR “Angola” OR “Benin” OR “Botswana” OR “Burkina Faso” OR “Burundi” OR “Cameroon” OR “Cape Verde” OR “Central African Republic” OR “Chad” OR “Comoros” OR “Congo” OR “Cote d Ivoire” OR “Ivory Coast” OR “Djibouti” OR “DRC” OR “Eritrea” OR “Ethiopia” OR “Gabon” OR “Gambia” OR “Ghana” OR “Guinea” OR “Kenya” OR “Lesotho” OR “Liberia” OR “Madagascar” OR “Malawi” OR “Mali” OR “Mauritania” OR “Mauritius” OR “Mayotte” OR “Mozambique” OR “Mocambique” OR “Namibia” OR “Niger” OR “Nigeria” OR “Réunion” OR “Rwanda” OR “Sahel” OR “Sao Tome and Principe” OR “Senegal” OR “Seychelles” OR “Sierra Leone” OR “Somalia” OR “South Africa” OR “RSA” OR “Sudan” OR “Swaziland” OR “Tanzania” OR “Togo” OR “Uganda” OR “Zambia” OR “Zimbabwe” | 136,671 |
| 17 | S15 OR S16 | 248,121 |
| 18 | S4 AND S11 AND S14 AND S17 | **1,286** |

| **Embase** – Searched on 01 Oct 2019 (2:14pm ET) | | |
| --- | --- | --- |
| **Number** | **Query** | **Results Returned** |
| 1 | (‘human immunodeficiency virus infection’/de OR ‘acute HIV infection’/de OR ‘Human immunodeficiency virus 1 infection’/de OR ‘Human immunodeficiency virus 2 infection’/de OR (‘HIV’ OR ‘Human Immunodeficiency Virus’ OR ‘Human Immunodeficiency Viruses’ OR ‘HIV infection’ OR ‘HIV infections’ OR ‘HIV/AIDS’ ):ab,ti) | 870,080 |
| 2 | (‘couple’/de OR ‘couples’/de OR ‘partner’/de OR ‘partnership’/de OR ‘relationship’/de OR ‘spouse’/de OR ‘married person’/de OR ‘married man’/de OR ‘married woman’/de OR ‘marriage’/de OR (‘couple’ OR ‘couples’ OR ‘partner’ OR ‘partners’ OR ‘partnership’ OR ‘partnerships’ OR ‘relationship’ OR ‘relationships’ OR ‘spouse’ OR ‘spouses’ OR ‘dyad’ OR ‘dyads’ OR ‘married’ OR ‘marital’ OR ‘marriage’ ):ab,ti) | 3,555,635 |
| 3 | (‘testing’/de OR ‘screening’/de OR ‘screening test’/de OR ‘counseling’/de OR ‘counselor’/de OR (‘testing’ OR ‘test’ OR ‘tests’ OR ‘tested’ OR ‘screen’ OR ‘screening’ OR ‘screenings’ OR ‘screened’ OR ‘counseling’ OR ‘counselling’ OR ‘counsel’ ):ab,ti) | 8,867,588 |
| 4 | 'africa south of the sahara'/exp | 649,914 |
| 5 | 'sub-saharan africa':ab,ti OR 'subsaharan africa':ab,ti OR 'sub saharan africa':ab,ti OR 'sub-sahara':ab,ti OR 'central africa':ab,ti OR 'southern africa':ab,ti OR 'east africa':ab,ti OR 'eastern africa':ab,ti OR 'west africa':ab,ti OR 'western africa':ab,ti OR 'angola':ab,ti OR 'benin':ab,ti OR 'botswana':ab,ti OR 'burkina faso':ab,ti OR 'burundi':ab,ti OR 'cameroon':ab,ti OR 'cape verde':ab,ti OR 'central african republic':ab,ti OR 'chad':ab,ti OR 'comoros':ab,ti OR 'congo':ab,ti OR 'cote d ivoire':ab,ti OR 'ivory coast':ab,ti OR 'djibouti':ab,ti OR 'drc':ab,ti OR 'eritrea':ab,ti OR 'ethiopia':ab,ti OR 'gabon':ab,ti OR 'gambia':ab,ti OR 'ghana':ab,ti OR 'guinea':ab,ti OR 'kenya':ab,ti OR 'lesotho':ab,ti OR 'liberia':ab,ti OR 'madagascar':ab,ti OR 'malawi':ab,ti OR 'mali':ab,ti OR 'mauritania':ab,ti OR 'mauritius':ab,ti OR 'mayotte':ab,ti OR 'mozambique':ab,ti OR 'mocambique':ab,ti OR 'namibia':ab,ti OR 'niger':ab,ti OR 'nigeria':ab,ti OR 'réunion':ab,ti OR 'rwanda':ab,ti OR 'sahel':ab,ti OR 'sao tome and principe':ab,ti OR 'senegal':ab,ti OR 'seychelles':ab,ti OR 'sierra leone':ab,ti OR 'somalia':ab,ti OR 'south africa':ab,ti OR 'rsa':ab,ti OR 'sudan':ab,ti OR 'swaziland':ab,ti OR 'tanzania':ab,ti OR 'togo':ab,ti OR 'uganda':ab,ti OR 'zambia':ab,ti OR 'zimbabwe':ab,ti | 352,637 |
| 6 | #4 OR #5 | 404,097 |
| 7 | #1 AND #2 AND #3 AND #6 | 8,533 |
| 8 | #1 AND #2 AND #3 AND #4 AND [embase]/lim | **3,909** |

| **PsycInfo** – Searched on 01 Oct 2019 (11:51pm) | | |
| --- | --- | --- |
| **#** | **Query** | **Results Returned** |
| 1 | DE (“HIV”) OR TI (“HIV” OR “human immunodeficiency virus” OR “human immunodeficiency viruses” OR “HIV infection” OR “HIV infections” OR “HIV/AIDS”) OR AB (“HIV” OR “human immunodeficiency virus” OR “human immunodeficiency viruses” OR “HIV infection” OR “HIV infections” OR “HIV/AIDS”) | 98,049 |
| 2 | DE (“couples” OR “sexual partners” OR “spouses” OR “dyads” OR “marriage”) OR TI (“couple” OR “couples” OR “partner” OR “partners” OR “partnership” OR “partnerships” OR “relationship” OR “relationships” OR “spouse” OR “spouses” OR “dyad” OR “dyads” OR “married” OR “marital” OR “marriage”) OR AB (“couple” OR “couples” OR “partner” OR “partners” OR “partnership” OR “partnerships” OR “relationship” OR “relationships” OR “spouse” OR “spouses” OR “dyad” OR “dyads” OR “married” OR “marital” OR “marriage”) | 1,570,207 |
| 3 | DE (“testing” OR “HIV testing” OR “screening” OR “screening tests” OR “counseling”) TI (“testing” OR “test” OR “tests” OR “tested” OR “screen” OR “screening” OR “screenings” OR “screened” OR “counseling” OR “counselling” OR “counsel”) OR AB (“testing” OR “test” OR “tests” OR “tested” OR “screen” OR “screening” OR “screenings” OR “screened” OR “counseling” OR “counselling” OR “counsel”) | 1,671,905 |
| 4 | TI (“sub-Saharan Africa” OR “Subsaharan Africa” OR “sub Saharan Africa” OR “sub-Sahara” OR “Central Africa” OR “Southern Africa” OR “East Africa” OR “Eastern Africa” OR “West Africa” OR “Western Africa” OR “Angola” OR “Benin” OR “Botswana” OR “Burkina Faso” OR “Burundi” OR “Cameroon” OR “Cape Verde” OR “Central African Republic” OR “Chad” OR “Comoros” OR “Congo” OR “Cote d Ivoire” OR “Ivory Coast” OR “Djibouti” OR “DRC” OR “Eritrea” OR “Ethiopia” OR “Gabon” OR “Gambia” OR “Ghana” OR “Guinea” OR “Kenya” OR “Lesotho” OR “Liberia” OR “Madagascar” OR “Malawi” OR “Mali” OR “Mauritania” OR “Mauritius” OR “Mayotte” OR “Mozambique” OR “Mocambique” OR “Namibia” OR “Niger” OR “Nigeria” OR “Réunion” OR “Rwanda” OR “Sahel” OR “Sao Tome and Principe” OR “Senegal” OR “Seychelles” OR “Sierra Leone” OR “Somalia” OR “South Africa” OR “RSA” OR “Sudan” OR “Swaziland” OR “Tanzania” OR “Togo” OR “Uganda” OR “Zambia” OR “Zimbabwe”) OR AB (“sub-Saharan Africa” OR “Subsaharan Africa” OR “sub Saharan Africa” OR “sub-Sahara” OR “Central Africa” OR “Southern Africa” OR “East Africa” OR “Eastern Africa” OR “West Africa” OR “Western Africa” OR “Angola” OR “Benin” OR “Botswana” OR “Burkina Faso” OR “Burundi” OR “Cameroon” OR “Cape Verde” OR “Central African Republic” OR “Chad” OR “Comoros” OR “Congo” OR “Cote d Ivoire” OR “Ivory Coast” OR “Djibouti” OR “DRC” OR “Eritrea” OR “Ethiopia” OR “Gabon” OR “Gambia” OR “Ghana” OR “Guinea” OR “Kenya” OR “Lesotho” OR “Liberia” OR “Madagascar” OR “Malawi” OR “Mali” OR “Mauritania” OR “Mauritius” OR “Mayotte” OR “Mozambique” OR “Mocambique” OR “Namibia” OR “Niger” OR “Nigeria” OR “Réunion” OR “Rwanda” OR “Sahel” OR “Sao Tome and Principe” OR “Senegal” OR “Seychelles” OR “Sierra Leone” OR “Somalia” OR “South Africa” OR “RSA” OR “Sudan” OR “Swaziland” OR “Tanzania” OR “Togo” OR “Uganda” OR “Zambia” OR “Zimbabwe”) | 79,107 |
| 5 | S1 AND S2 AND S3 AND S4 | **1,056** |

| **PubMed** – Searched on 01 Oct 2019 (2:17pm ET) | | |
| --- | --- | --- |
| **Number** | **Query** | **Results Returned** |
| 1 | HIV[MeSH Terms] OR HIV[Text Word] OR Human Immunodeficiency Virus[Text Word] OR Human Immunodeficiency Viruses[Text Word] OR HIV/AIDS[Text Word] OR HIV infection[Text Word] OR hiv infections[MeSH Terms] OR hiv infections[Text Word] | 394967 |
| 2 | couple[Text Word] OR couples[Text Word] OR partner[Text Word] OR partners[Text Word] OR partnership[Text Word] OR partnerships[Text Word] OR relationship[Text Word] OR relationships[Text Word] OR spouse[Text Word] OR spouses[MeSH Terms] OR spouses[Text Word] OR dyad[Text Word] OR dyads[Text Word] OR married[Text Word] OR marital[Text Word] OR marriage[MeSH Terms] OR marriage[Text Word] | 2059367 |
| 3 | testing[Text Word] OR test[Text Word] OR tests[Text Word] OR tested[Text Word] OR screen[Text Word] OR screening[Text Word] OR screenings[Text Word] OR screened[Text Word] OR counseling[MeSH Terms] OR counseling[Text Word] OR counselling[Text Word] OR counsel[Text Word] | 4243061 |
| 4 | Africa south of the sahara[MeSH Terms] OR Africa South of the Sahara[Text Word] OR sub-Saharan Africa[Text Word] OR Subsaharan Africa[Text Word] OR sub Saharan Africa[Text Word] OR sub-Sahara[Text Word] OR Central Africa[Text Word] OR Southern Africa[Text Word] OR East Africa[Text Word] OR Eastern Africa[Text Word] OR West Africa[Text Word] OR Western Africa[Text Word] OR Angola[Text Word] OR Benin[Text Word] OR Botswana[Text Word] OR Burkina Faso[Text Word] OR Burundi[Text Word] OR Cameroon[Text Word] OR Cape Verde[Text Word] OR Central African Republic[Text Word] OR Chad[Text Word] OR Comoros[Text Word] OR Congo[Text Word] OR Côte d’Ivoire[Text Word] OR Ivory Coast[Text Word] OR Djibouti[Text Word] OR DRC[Text Word] OR Eritrea[Text Word] OR Ethiopia[Text Word] OR Gabon[Text Word] OR Gambia[Text Word] OR Ghana[Text Word] OR Guinea[Text Word] OR Kenya[Text Word] OR Lesotho[Text Word] OR Liberia[Text Word] OR Madagascar[Text Word] OR Malawi[Text Word] OR Mali[Text Word] OR Mauritania[Text Word] OR Mauritius[Text Word] OR Mayotte[Text Word] OR Mozambique[Text Word] OR Mocambique[Text Word] OR Namibia[Text Word] OR Niger[Text Word] OR Nigeria[Text Word] OR Réunion[Text Word] OR Rwanda[Text Word] OR Sahel[Text Word] OR Sao Tome[Text Word] OR Principe[Text Word] OR Senegal[Text Word] OR Seychelles[Text Word] OR Sierra Leone[Text Word] OR Somalia[Text Word] OR South Africa[Text Word] OR RSA[Text Word] OR Sudan[Text Word] OR Swaziland[Text Word] OR Tanzania[Text Word] OR Togo[Text Word] OR Uganda[Text Word] OR Zambia[Text Word] OR Zimbabwe[Text Word] | 448764 |
| 5 | #1 AND #2 AND #3 AND #4 | **3,914** |
