# Supplementary material for: Strategies to increase couples HIV testing and counselling in sub‐Saharan Africa: a systematic review
Source: J Int AIDS Soc. 2023 Mar 16;26(3):e26075. doi: 10.1002/jia2.26075 (PMC10020817; doi:10.1002/jia2.26075)
Supplement: Supplementary file 2 — Supplementary information [file JIA2-26-e26075-s002.docx]

**Supplementary Table.** Summary of 30 published articles included in the systematic review for couples HIV counseling and testing, 1993-2019

| **Citation** | **Main strategy being tested** | | **Number/proportion of CHTC uptake** | | **Effect size (if applicable)** | | **Study Summary** |
| --- | --- | --- | --- | --- | --- | --- | --- |
| **Home-based CHTC (7 studies)** | | | | | | | |
| Becker, et al., 2014 | New testing location (home) | 145/167 (87%) | | N/A | | Offering CHTC along with couple family planning services to couples in their homes resulted in 87% acceptance of CHTC. | |
| Dalal, et al., 2013 | New testing location (home) | 38% Kibera  52% Lwak | | N/A | | 24,450 people were offered door-to-door home-based testing and counseling, 19,966 (81.7%) accepted testing. Among individuals reporting being in a married or cohabitating relationship, 38.0% in Kibera (urban setting) and 51.7% in Lwak (rural setting) chose CHTC. | |
| Doherty, et al., 2013 | New testing location (home)  Standard of care | 216/1031 (21%) New testing location  76/801 (10%) Standard of care | | Prevalence ratio: 2.24 [95% CI 1.49 to 3.03]. | | In intervention clusters (communities), lay counsellors conducted door to door outreach and offered home based HIV counselling and testing to anyone over 14 years. Control clusters had the standard of care (HIV counselling and testing services at local clinics). The intervention had positive effects on couple counselling and testing, with double the prevalence of couple counselling and testing in the intervention arm (21%) compared with the control arm (10%). | |
| Krakowiak, et al., 2016 | “HOPE”: home-based education and HIV testing  “INVITE”: written invitations for male partners to attend clinic | 192/248 (77%) HOPE  62/254 (24%) INVITE | | RR 3.17  [95% CI 2.53-3.98] | | Women attending their first antenatal visit (n= 601) were randomized to home-based education and HIV testing within 2 weeks of enrollment (HOPE) or to written invitations for male partners to attend clinic (INVITE). Couples in the HOPE arm (77%) were 3 times as likely to have been tested as a couple as the INVITE arm (24%) | |
| Matovu, et al., 2002 | New testing location (home) | 1149/6765 (17%) | | N/A | | Blood draws were collected as part of an epidemiological survey in participants homes. Individuals received pre-test counselling and could request their HIV test results as individuals or couples. 17% (1149 / 6765) of those who received results were counselled as a couple. | |
| Osoti, et al., 2014 | New testing location (home)  Written invitations for male partners to come to clinic | 128/150 (85%) home-based testing  54/150 (36%) clinic-invitation | | RR 2.37  [95% CI 1.90-2.96] | | Women attending their first ANC visit who were unaccompanied by their male partners (n=300) were randomized to home visits or written invitations for male partners to come to clinic. CHTC was significantly higher in home-visit than clinic-invitation arm (n=128, 85% vs n=54, 36%; p<0.001). | |
| Tumwebaze, et al., 2012 | New testing location (home), facilitated mutual disclosure during post-test counseling | 164/544 (30%) | | N/A | | Community outreach workers (an existing health cadre in Uganda) approached all households to notify that home-based HIV counseling and testing would be offered in the area. During visits, outreach workers provided education, obtained consent, and offered Individual HIV counselling and testing. Post-test counselling was performed, and a card with HIV status was offered to facilitate establishment of care at local HIV clinics. Couples were offered facilitated mutual disclosure of HIV test results. 544 adults reported a partner in the home and 164 couples were tested with 161 disclosing results. | |
| **Enhancing the clinic environment (4 studies)** | | | | | | | |
| Courtenay-Quirk, et al., 2018 | Clinic enhancements – tuberculosis clinics | | 35% Overall  Cluster 1: 105/280 (37.5%)  Cluster 2*: 101/324 (31.2%)  *delayed 3 months | | N/A | | Within tuberculosis care settings, improving the clinic environment (i.e., record keeping of HIV service delivery, training for clinic staff and peer education volunteers on CHTC) led to an increase in CHTC among newly diagnosed TB patients in both clusters from 1.8% (pre-implementation) to 35.2% (post-implementation). |
| Homsy, et al., 2006 | Clinic enhancements – antenatal clinics and maternity wards | | 102/3695 (2.8%) ANC  260/698 (37%) Maternity wards | | N/A | | Opt-out routine HIV counseling and testing services were established in ANC and the maternity ward. Couples counseled together represented 2.8% of all persons tested in the ANC and 37% of all persons tested in the maternity ward. |
| Kababu, et al., 2018 | Clinic enhancements (Counsellor-supported disclosure model) | | 36/127 (28.4%) Counsellor-supported disclosure  11/149 (7.4%) Control | | aOR 8.01  [95% CI 2.75-23.32] | | The counsellor-supported disclosure model used HIV testing counsellors to empower the index HIV testing client (individual presenting alone for testing; n=276) to invite their sexual partner for CHTC at the clinic and linking the couples to appropriate post-HIV test interventions. It also included SMS and phone follow-up. Counselling included five main topics: i) HIV information including discordance and concordance; ii) couple communication including effective partner communication, iii) benefits of disclosure and possible methods of disclosure, iv) assertive and negotiations skills, and v) self-awareness skills. Participants in the intervention arm of the study had eight times higher odds of taking up CHTC (28%) compared to their control counterparts (7%). |
| Lyatuu, et al., 2018 | Clinic enhancements, community recruitment | | 1463/4260 (36%) Intervention sites  5414/ 30950 (18.3%) Control sites | | p = 0.07 | | This study integrated initiatives to increase male partner participation in routine antenatal care in six health facilities. Initiatives included offering couple friendly services at antennal clinics and promoting male partner participation through community activities via community leaders. After one year, couple HIV testing in the six intervention sites had tripled from 11.9% at base- line to 36.0% (p<0.001) while there was very little change (from 17.7% to 18.3%) in the 203 control sites (p = 0.07). |
| **Distribution of HIV self-test kits (4 studies)** | | | | | | | |
| Gichangi, et al., 2018 | Standard-of-care and a standard information card  An improved card stating the importance of male HIV testing  Self-test kits and information | | 110/406 (27%) Standard information card  136/387 (35.1%) Improved information card  334/422 (79%) Self-test kits | | Improved card*: aOR 1.48  [95% CI 1.07-2.05]  Self-test kits*: aOR 11.52  [95% CI 8.03-16.53]  *reference group: standard information card | | In this three-arm randomized control study, consenting women attending the first antenatal visit at one of 14 facilities were randomized to one of three arms (n=1410): standard information card; improved information card (stating the importance of male HIV testing); or two oral HIV self-test kits with the improved invitation arm. Women in the arm who received HIV self-test kits had significantly greater odds (aOR 11.5) of reporting couple HIV testing (79%) compared to those receiving standard of care (27%). Women in the arm who received an improved card also had significantly greater odds (aOR 1.5) of reporting couple HIV testing (35%) compared to those receiving standard of care but not to the same extent as those receiving self-test kits. |
| Masters et al., 2016 | Standard-of-care and invitation card for male partners  HIV self-testing kits | | 33.2% Standard-of-care and invitation card for male partners  75.4% HIV self-testing kits | | RR 2.27 (1.90–2.71), p < 0.001 | | This randomized trial in Kisumu, Kenya, randomized six hundred antenatal and postpartum women aged 18–39 years were to an HIV self-testing (HIVST) group or a comparison group. Participants in the HIVST group were given two oral HIV test kits, instructed on how to use them, and encouraged to distribute a test kit to their male partner or use both kits for testing as a couple. Participants in the comparison group were given an invitation card for clinic-based HIV testing and encouraged to distribute the card to their male partner, a routine practice in many health clinics. Couples testing was more likely in the HIVST group than the comparison group (75.4% versus 33.2%, difference = 42.1%, 95% CI 34.7% to 49.6%, *p* < 0.001). In this study, the self-testing intervention was safe and effective at promoting partner HIV testing and couples testing. |
| Pintye, et al., 2019 | HIV self-testing kits | | 358/362 (99%) | | N/A | | This study offered self-tests for at-home couples or partner HIV testing to HIV-uninfected women seeking routine maternal and child health and family planning services at eight facilities in Kisumu, Kenya. Overall, 3620 women were offered self-tests for at-home male partner HIV testing. Approximately one-quarter (24%) of all women offered self-tests accepted. Overall, 1422 (39%) women reported having a partner of unknown HIV status, of whom 755 (53%) accepted self-tests. |
| Thirumurthy, et al., 2016 | HIV self-testing kits | | 27/53 (51%) Antenatal patients  62/91 (68%) Postpartum patients    53/64 (83%) drop-in center for female sex workers | | N/A | | This study provided multiple self-tests to women at high risk of HIV acquisition in order to promote partner HIV testing and facilitate safer sexual decision-making. HIV-uninfected women were recruited at two sites in Kisumu, Kenya: a health facility with antenatal (ANC) and postpartum (PPC) clinics, and a drop-in center for female sex workers (FSW). Most index participants with a primary sexual partner distributed a self-test to that partner (53/58, 91% ANC; 91/106, 86% PPC; 64/85, 75% FSW). Among self-tests distributed to and used by IPs’ primary sexual partners, couples testing occurred frequently (27/53, 51% ANC; 62/91, 68% PPC; and 53/64; 83% FSW). |
| **Verbal or written invitations (4 studies)** | | | | | | | |
| Becker, et al., 2010 | Verbal encouragement, written letter | | 119/760 (16%) | | N/A | | During antenatal care, 760 women were randomized to the CHTC arm (instead of an individual testing study arm), asked to bring their husbands with them for CHTC at the next antenatal clinic visit, and given a letter for the husband which did not mention CHTC. This resulted in 135 couples tested with 119 (16%) receiving results. |
| Heyward, et al., 1993 | Verbal encouragement | | 2.2% | | N/A | | 187 HIV-positive and 177 HIV-negative women received pre- and post-test counseling. Only 2.2% of women returned with their partner for joint HIV counseling and testing. |
| Jefferys, et al., 2015 | Written letter/invitation for male partner (ANC) | | 138/170 (81%) | | N/A | | Women attending ANC for the first time during their current pregnancy were recruited into the study from three health centers. Women were given a written invitation letter for their male partner requesting their presence at the next routine antenatal clinic visit (letter did not state that an HIV test would be offered). Out of 318 women who received an invitation letter for their partner, 53.5 % returned with their partners for a joint antenatal clinic session; of these, 81% proceeded to CHTC. (n=138 couples). Male attendance varied significantly between the rural and urban study sites (p < 0.001) with rates as high as 76 % at the rural site compared to 31 % at the urban health center. |
| Theuring, et al., 2016 | Written letter/invitation for male partner  Verbal encouragement to invite male partner | | 30/97 (30.9%) written letter  28/102 (27.5%) verbal encouragement | | OR 1.2  (p=0.59) | | Pregnant women attending antenatal care without partners received official letters inviting the partner to attend future visits. A control group was instructed to verbally invite partners. Rates for male partner return, couple voluntary counselling and testing (CVCT), and influencing factors were analyzed. From 199 antenatal care clients in total, 97 were assigned to the invitation letter group; 30 of these (30.9%) returned with their male partners for ANC. In the control group of 102 women, 28 (27.5%) returned with their partner. Written and verbal invitations had comparable outcomes. |
| **Community agents/recruiters (3 studies)** | | | | | | | |
| Allen et al. 2007 | community recruiters, written letter/invitation | | 1411/9900 (14.3%) | | N/A | | Over four months, “Influence Network Agents (INAs)” from various sectors (health, religious, community) distributed 9,900 invitations, leading to 14.3% uptake of CHTC |
| Lambdin, et al., 2011 | Influence network agents, performances, media | | 2409/3414 (71%) Kitwe  3212/4274 (75%) Ndola | | N/A | | This study evaluated the effectiveness of several promotional strategies to increase demand for CHTC in Kitwe and Ndola, Zambia. The program attracted more than 7,600 couples through the use of radio broadcasts, billboards, and other strategies. The most effective recruiting technique was the use of local residents trained as “influence agents” to reach out to friends, neighbors, and others in their sphere of influence. |
| Wall, et al., 2012a  Wall, et al., 2012b | Influential network leaders and agents who in conducted community- based promotion and distribution of CVCT invitations (primary intervention), a mobile unit, transport reimbursement, lunch, and childcare | | 1727/29119 (6%) Zambia  4513/24991 (18%) Rwanda | | N/A | | Influential network leaders and agents made public endorsements of CHTC in Rwanda and Zambia. Public endorsements conducted by influence network agents (INLs) included priests/pastors discussing CHTC during sermons, senior nurses giving talks to clinic patients, factory foremen encouraging employees to test with their spouses, or parent-teacher association leaders presenting CHTC at local meetings. Invitations were delivered directly to couples/individuals and through community and health facilities that detailed CHTC facility directions and procedures. Couples attending CHTC services received transport reimbursement, lunch, and childcare at CVCT sites. Invitations distributed by influential people were successful in prompting couples to seek joint HIV testing. |
| **Index Partner Tracing (2 studies)** | | | | | | | |
| Rosenberg, et al., 2015 | Written invitation/letter + partner tracing by community health worker  Written letter/invitation | | 74/100 (74%) Written invitation plus partner tracing  52/100 (52%) Written invitation | | Risk difference 22% [95% CI 9–35; p=0·001] | | Women were randomly assigned (1:1) to either the invitation only group or the invitation plus tracing group with block randomization. In the invitation only group, women were provided with an invitation for male partners to present to the antenatal clinic. In the invitation plus tracing group, women were provided with the same invitation, and partners were traced if they did not come to the health facility for HIV testing. A community health worker contacted the male partner in-person or over phone with tracing info provided by the female partner. The community health worker used messages consistent with the invitation and strategies to help participants overcome barriers to attendance and then agree on a day and time to attend. The invitation did not disclose the index’s HIV-positive status or mention CHTC. After 1 month, 74 couples in the invitation plus tracing group and 52 in the invitation only group presented to the clinic and had CHTC (risk difference 22%, 95% CI 9–35; p=0·001) |
| Joseph-Davey, et al., 2019 | Track and trace partners and children of index patients living with HIV | | 360/911 (40%) | | N/A | | This study evaluated the impact of implementing index partner/child testing, in which clinic staff were trained to trace partners and children of women living with HIV, either recently diagnosed or on antiretroviral therapy (ART). These services were implemented in public health hospitals and clinics in South Africa. During index patient post-HIV test counselling or ART adherence counselling (for patients already on ART), counsellors and nurses asked index patients to refer their partner or children who did not know their HIV status or were not known to be on ART for testing. The provider would give the index patient three options for testing of their partner/child: written invitation letter; phone call from the clinician; or the index patient returned for testing with their partner or child. Overall 40% of index patients were counselled and tested together with their partner via CHTC. |
| **Relationship strengthening/intensive couple counseling (2 studies)** | | | | | | | |
| Darbes, et al., 2019 | Multi-session couples counseling | | 71/168 (42%) couple counseling  20/164 (12%) SOC | | p<0.001 | | 334 couples recruited from the community (eg, markets and community events) who had not disclosed their HIV status to one another. Intervention couples received one couples-based group session and four couples counseling sessions covering communication skills, intimate partner violence, and HIV prevention. Intervention couples were significantly more likely to have participated in CHTC [42% vs. 12%]. |
| Turan, et al., 2018 | New testing location (home) + multi-session couples counseling  Written invitation/letter for male partner (SOC) | | 34/53 (64%) home-based CHTC and intensive counseling  12/52 (23%) Written invitations (SOC) | | RR 2.78  [95% CI 1.63-4.75] | | This intervention aimed to promote couple collaboration in health during pregnancy and postpartum in southwestern Kenya. Pregnant women were randomized to intervention (n=64) or standard care (n=63) arms. In the intervention arm, lay health workers conducted three couple home visits, including health education, couple relationship and communication skills, and offers of couple HIV testing and counseling (CHTC) services. At follow-up, women in the intervention arm had almost three times higher RR for undergoing CHTC with their male partner during the study period, compared with women in the standard care arm (RR = 2.78; 95% confidence interval: 1.63–4.75) |
| **Incentives (1 study)** | | | | | | | |
| Sibanda, et al., 2015 | Financial incentives for CHTC  Standard mobilization | | 7852/14099 (55.70%) incentives for CHTC  1062/10580 (10%) Standard mobilization (comparison) | | aOR 13.5  [95% CI 10.5-17.4] | | 68 rural communities (clusters) with mobile HIV testing services were randomly assigned to receive or not receive incentives for CHTC. In intervention communities, couples who tested together could select one of three grocery items worth US$1·50. Standard mobilization for testing was done in comparison communities. 7852 (55·7%) testers in the intervention group versus 1062 (10·0%) in the comparison group tested with a partner (adjusted odds ratio 13·5 [95% CI 10·5–17·4]). |
| **Group education with invitation coupons (1 study)** | | | | | | | |
| Matovu, et al., 2016 | Small group couple and male-focused interactive sessions, received invitation coupons for CHTC  General adult health education sessions | | 104/697 (20.3%) Group sessions and coupon  54/477 (13.7%) General adult health education sessions | | Adjusted prevalence ratio: 1.43 [95% CI 1.02-2.01], P = 0.04 | | This study randomly assigned six clusters (1:1) in rural Uganda to receive the intervention or serve as the comparison arm. In the intervention clusters, individuals attended small group, couple and male-focused interactive sessions, reinforced with testimonies from ‘expert couples,’ and received invitation coupons to test together with their partners at designated health facilities. In the comparison clusters, participants attended general adult health education sessions but received no invitation coupons. Couples’ HCT uptake was higher in the intervention than in the comparison clusters (20.3% versus 13.7%). |
| **Offering HIV testing for male partners at other community venues (1 study)** | | | | | | | |
| Ditekemena, et al., 2011 | New testing location: church  New testing location: bar | | Overall: 208/591 (35%)  86/189 (46%) church venue  89/236 (38%) Bar venue  33/166 (20%) Health center (SOC) | | Church*: OR 3.36 [95% CI: 2.08–5.42 (P<0.001)  Bar*: 2.44 [95% CI: 1.53 – 3.87 (p<0.001)  *Reference: health center | | The primary outcome of the trial was male participation (of pregnant women receiving antenatal-based HIV testing) in HIV testing after randomization to one of three venues (health center, church, and bar). Of the 591 men who were tested at one of the venues and invited for couple counselling with their female partners, 208 (35%) men presented for couple counselling. The attendance at the church venue was 46%, over three times greater than at the health center venue (20%). Couple counselling at the bar venue was 38%, more than double the rate at the health center venue. |

CHTC = couples HIV testing and/or counseling
